# Integrating Model Development Across Computational Neuroscience, Cognitive Science and Machine Learning

**DOI:** 10.1016/j.neuron.2023.03.037

**Published:** 2023-04-25

**Authors:** Padraig Gleeson, Sharon Crook, David Turner, Katherine Mantel, Mayank Raunak, Ted Willke, Jonathan D. Cohen

**Affiliations:** Department of Neuroscience, Physiology and Pharmacology, University College London; School of Mathematical and Statistical Sciences, Arizona State University; Princeton Institute for Computational Science & Engineering, Princeton University; Princeton Neuroscience Institute, Princeton University; Intel Labs, Intel Corp; Princeton Neuroscience Institute, Princeton University

## Abstract

Neuroscience, cognitive science, and computer science are benefitting increasingly through their interactions. This could be accelerated by direct sharing of computational models across disparate modeling software used in each. We describe a Model Description Format designed to meet this challenge.

## Background

Research in neuroscience, cognitive science, and the areas of artificial intelligence (AI) and machine learning (ML) in computer science has long shared common questions concerning the mechanisms underlying intelligence, and progress in each field has been driven profoundly by interactions with the others. For example, McCulloch and Pitts^1^, as well as Hebb^2^, were inspired in their work on the brain by that of Turing in defining the principles of computation, and in turn, their work inspired Rosenblatt’s Perceptron. Work in neuroscience and psychology provided the foundations for breakthroughs in ML for reinforcement learning, which in turn transformed our understanding of neuromodulatory mechanisms such as the brain’s dopamine system, while the development of the backpropagation learning algorithm and convolutional neural networks, jointly by psychologists and computer scientists, provided the foundation for the explosion of modern work on deep learning. Most recently, insights into specific brain subsystems (such the hippocampus) have inspired the development of new forms of external memory (such as the Neural Turing Machine), while, conversely, the development of algorithms for recurrent neural networks has guided models of how working memory is implemented in brain structures such as the prefrontal cortex and basal ganglia.

We are now at a critical juncture. On the one hand, as suggested above, neuroscience and cognitive science have generated mechanistically explicit models of critical brain subsystems, as well as many higher level cognitive functions. At the same time, machines designed by computer scientists now match or surpass humans in tasks long considered to be the unique province of the human brain, such as visual object recognition and game playing (e.g., Jeopardy, chess, Go), and are making rapid strides in natural language processing. On the other hand, despite this progress, the flexibility and efficiency of processing in the human brain remain unique. No existing machine exhibits the scope of capabilities that are as diverse as an adult human, or the ability to acquire these through training, no less with the efficiency or processing that it exhibits (while relying on approximately 20 watts of power). Understanding how the brain achieves this, and how it can be achieved in artificial form, remains one of the greatest challenges facing science and technology. It seems inevitable that, as in the past, progress will require the fluid exchange of ideas, discoveries, and methods across the relevant disciplines. However, the dramatic increase in sophistication of modeling efforts required to address this challenge has brought with it a new set of issues that threaten the exchange of innovations and advances across these disciplines, and thereby their continued potential for transformative impact.

## Current Obstacles to Model and Knowledge Exchange

Historically, the exchange of ideas across scientific disciplines in a precise form has relied on mathematical expressions that are readily interpretable. However, as the focus of research has progressed to increasingly complex mechanisms and capabilities, progress has come to rely increasingly on numerical methods — that is, computational modeling. This poses two closely related, and rapidly increasing, challenges to the acceleration of progress: i) *siloing of communities*: the proliferation of domain-specific software tools for building, executing and analyzing increasingly complex computational models; and ii) *hardware optimization*: the need to make the execution of such models tractable at scale.

### Siloing of communities

Reflecting a “Tower of Babel” problem, many of the powerful software tools for constructing and executing computational models have been developed independently, within and across different (sub)fields, posing substantial barriers to the exchange of such technology and the insights that can emerge from them^3^. For example, investigators interested in how response properties of individual neuronal cell types may impact learning at the circuit level must choose between developing their models in neuroscience-specific environments such as NEURON [https://neuron.yale.edu/neuron] that support the creation of biophysically realistic models of neuronal function, and ones from ML such as PyTorch [https://pytorch.org] and TensorFlow [https://tensorflow.org] that support computationally efficient network-level algorithms for learning. At present there are no automated means for translating models between these environments. Understandably, this has been driven by differences in phenomena of interest, levels of analysis, and approaches to modeling. However, computational approaches and tools developed in one domain or for one purpose often are found to have fundamental value in others (e.g., Bayesian methods, deep learning). Similarly, advances in the tools for developing and evaluating models (e.g., hyper-parameterization and data fitting) are proving useful across domains. Unfortunately, the development of software tools that exploit this convergence has not kept pace. Some widely used tools have emerged for modeling in particular domains (e.g., NEURON for computational neuroscience and PyTorch and TensorFlow for ML). However, rarely do tools created for computational modeling in one domain interoperate with those in other domains, even when the fundamental constructs — and the insights that can be gained from them — are closely related or the same. This imposes inefficiencies at best; and, at worst, it obscures opportunities for sharing ideas and technologies that can generate new insights and drive progress. Even where there are close parallels in the approaches and tooling needs across domains, often each uses its own application-specific software, making it difficult to see shared opportunities; and, even where those are recognizable, translating individual models or adapting tools developed in one environment for use in another is not considered to be worth the cost. Anyone involved in computational modeling is painfully familiar with the burden of porting code for a model from another laboratory (and sometimes even from individuals within their own laboratory!), often requiring translation into a different language, and/or lacking clear documentation. At best this costs many hours of effort, and at worst reimplementation is prone to errors and corresponding replication failures. Furthermore, when deemed worthwhile, such efforts usually focus on translating a specific model between two particular environments, which does not solve the problem for other models or environments. Creating a means by which models generated in one software environment can be automatically translated into a common format, examined, validated, and then shared with and/or integrated into other environments would have a transformative impact on the ability for interdisciplinary interactions and cross-fertilization going forward, as discussed further below.

### Hardware optimization

As models become increasingly sophisticated, whether to accommodate the complexity of mechanisms in the brain, and/or to address the complexity of functions required to achieve natural intelligence in machines, the need for optimization of execution (ensuring the model runs as efficiently as possible in the chosen software/hardware environment) has become critical. This, in turn, has carried with it the “Tower of Babel” problem described above: tools for optimization are typically specialized for specific software and/or hardware environments, presenting a barrier to their use with others.

One approach to this has been the development of “intermediate representations” (IRs) — standard formats into which models created using higher level software tools (such as PyTorch and TensorFlow) can be translated, and then optimized for different hardware platforms. For example, this is the approach being taken with formats such as ONNX (Open Neural Network Exchange; https://onnx.ai). However, such efforts have been designed largely for the optimization of applications in ML, and are not as easily accessed by or adapted to environments used in other domains, such as NeuroML^4^ in neuroscience, or ACT-R^5^ in cognitive science. Other approaches that take a more general purpose approach (such as LLVM; https://llvm.org) are usually at too low a level to be practical for those constructing computational models in neuroscience and cognitive science. Limitations in the number and effectiveness of tools for acceleration of models developed in those scientific disciplines, and the expertise required to use them, has substantially limited the scope and sophistication of such modeling efforts, and their interactions with similar efforts in AI and ML. The ability to translate models created in those domains into a standard format, and from there into existing IRs, would open up a tremendous opportunity for neuroscientists and cognitive scientists to leverage the considerable efforts being put into optimization by the ML community. A fully general standard would also open other novel opportunities for optimization. Existing ML IRs have targeted traditional computing hardware, such as CPUs and GPUs, but have not yet exploited emerging technologies, such as quantum computing. Such hardware may have particular value for a broad class of models that — complementary to those that use deep learning methods — address the dynamics of constraint satisfaction and decision making (see below). Such models often involve highly interdependent, fine-grained interactions among processing units and multiple time scales of interaction, factors that are not addressed by current approaches in AI or ML. They are also not well served by standard techniques such as loop vectorization, and may be more effectively addressed by novel (e.g. polyhedral) approaches to optimization and hardware implementation (e.g. quantum approaches). These, in turn, may be most suitable for a graph-based format that preserves access to higher-level model structure and operations.

## A Standardized Model Description Format

**Figure 1:**
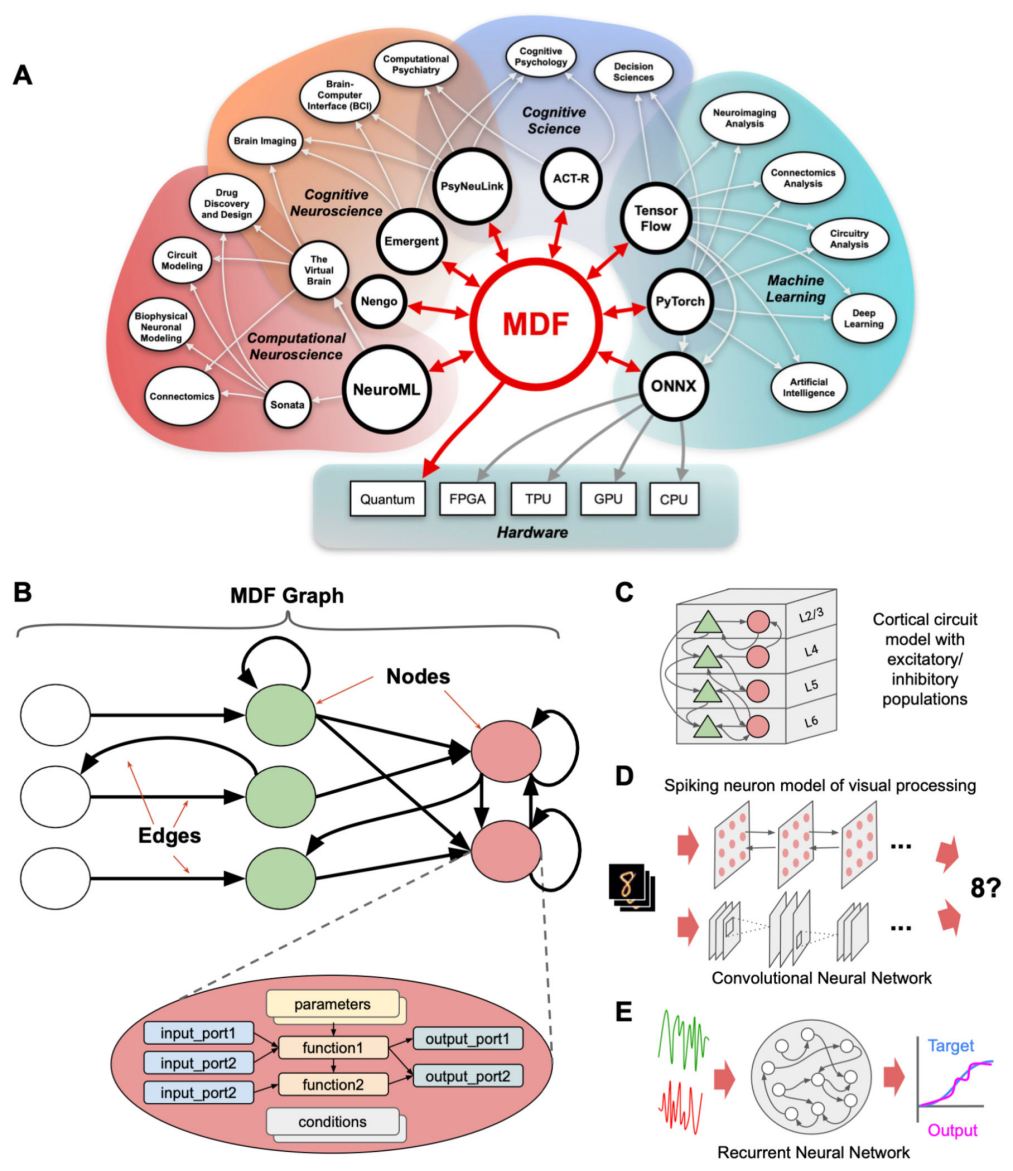
A) Overview of potential scope of ModECI effort, with MDF as a “hub” that allows the translation and exchange of computational models over “spokes” that connect to modeling environments at multiple levels of analysis and in diverse domains. B) Schematic example of MDF showing a model represented as a graph, in which nodes represent computational elements, edges the transmission of data and flow of execution. Inset shows internal components of a node. C,D,E) Examples of models at different levels of analysis and of different forms, all of which can be represented using MDF.

The goal of enhancing interactions of computational neuroscientists and cognitive scientists with ML and AI researchers prompted the formation of the Model Exchange and Convergence Initiative (ModECI; https://modeci.org). The aim of this effort is to define, implement and maintain a standard Model Description Format (MDF; https://github.com/ModECI/MDF) that can be used to exchange models across disparate modeling software environments in machine readable form, and/or lower them to IRs for machine optimization, in a manner that fully preserves a model’s structure and functionality. Upon formation, ModECI carried out a series of workshops to engage with and get direction from the diverse communities it is intended to serve. This involved representatives from academia, industry, and non-profit organizations devoted to the support of open science and interdisciplinary exchange (https://modeci.org/#communityPage). These workshops reaffirmed the potential value of developing an MDF and provided a clear set of desiderata, priorities, and challenges for its development, outlined here. 1) The effort should focus specifically on translation between existing programming and execution environments (Fig. 1A), and not the creation of a new, general purpose simulation and/or programming environment. 2) Accordingly, the format should prioritize model translation (in a way that preserves model structure) over efficiency of execution — that is, *transpilation* rather than *compilation* — a tradeoff that favors the expression of a model as a computational graph. 3) The format should support arbitrary patterns of control flow, which presents a challenge for standard forms of computational graphs. 4) The initial focus should be on standardizing the specification of the structure and execution of the model itself, rather than procedures for its use (e.g., parameterization, training protocols, data-fitting), with the latter as a subsequent target for further development.

Based on these considerations, ModECI contributors have developed an initial specification for MDF (https://mdf.readthedocs.io/specification). This expresses models in a serialized format as a computational graph (Fig. 1B), in which **nodes** carry out mathematical operations, with **ports** that are used to receive input for those operations and provide the output to other nodes, and **edges** that transmit information between ports on different nodes. The formal specification of the format includes a control flow syntax that can specify arbitrary orders of node execution (based on defined **conditions**), including nodes with different times scales of computation, cyclic graphs, and hierarchically structured patterns of execution. The standard is augmented with a function ontology (https://mdf.readthedocs.io/functions) that includes all non-conditional operators in the ONNX function library (https://onnx.ai/onnx/operators), and can be extended with additional functions that may be of broad use or of use within specific scientific domains (e.g. FitzHugh Nagumo integrator in neuroscience or the drift diffusion integration in cognitive science). MDF also has detailed online documentation (https://mdf.readthedocs.io), and an open source Python library of tools (https://github.com/ModECI/MDF) that includes a reference importer and exporter of models serialized in JSON, YAML or binary formats, visualization tools for structural validation, and an execution engine that can be used to validate expected behavior of models. This Python package is intended to be used as the basis for other libraries that employ MDF for the automated exchange of models across existing tools for the analysis, optimisation, and visualization of models from multiple disciplines.

MDF supports specification of models at many levels of analysis (Fig. 1C-E), from biophysically detailed individual neurons and synapses, to neural populations, and abstract tensor-based models, such as those widely used in ML. To demonstrate this flexibility, prototype interpreters have been developed, with corresponding demonstration examples that translate models between MDF and widely used domain-specific exchange formats (e.g. NeuroML in neuroscience and ONNX in ML), as well as model design and execution environments (e.g., PyTorch in ML).

## Conclusion and future work

The current version of MDF, while functional, should still be considered a prototype intended to highlight the practicality and potential of such a format, and to serve as a foundation for further development. ModECI, responsible for the continued development of MDF, is an effort in support of an open-source, community-driven software project. Current efforts are focused on maturing existing interfaces between MDF and widely used resources, such as NeuroML and PyTorch, broadening its scope to include others (such as The Virtual Brain^6^, Emergent^7^, Nengo^8^, ACT-R^5^, and TensorFlow), expanding MDF capabilities to include the specification of model execution environments (such as training and hyper-parameterization protocols), and interfacing with data sharing efforts such as Neurodata Without Borders^9^ and the Brain Imaging Data Structure, BIDS^10^. A guide to the current status of the specification, the prototype implementation, supported import/export environments and examples of models and datasets in the format can be found here: https://modeci.org/quickstart.

ModECI welcomes the participation of anyone interested in these efforts to broaden the capabilities and scope of MDF and, more generally, its goal of promoting the fluid exchange of models across levels of analysis and disciplines involved in computational modeling of the functioning of the human brain, and reproducing its capabilities in synthetic systems. In these ways, ModECI promises not only to provide a medium for interdisciplinary research, but also to serve as a model for how this can be effectively pursued in a community-based manner.
